# Effect of a positive thinking skills training program on psychological adjustment among psychiatric patients’ caregivers

**DOI:** 10.1186/s12888-025-07276-3

**Published:** 2025-10-23

**Authors:** Nahed Hafez Abd El-Hay, Amal Sobhy Mahmoud, Abeer El-Sayed Berma

**Affiliations:** 1 Department of Psychiatric Nursing and Mental Health, Faculty of Nursing, Port Said University, Port Said, Egypt; 2 Department of Psychiatric Nursing and Mental Health, Faculty of Nursing, Misr University for Science and Technology(MUST), Giza, Egypt

**Keywords:** Caregivers, Positive thinking, Psychiatric illness, Psychological adjustment

## Abstract

**Background:**

Psychiatric disorders are becoming a major public health problem. The prolonged caregiving responsibilities for psychiatric patients can deplete the family’s energy, resulting in exhaustion and increased negative emotions, such as feelings of despair, guilt, depression, and helplessness, and a significant decrease in their psychological adjustment which requires adequate support and training.

**Aim:**

The present study aims to explore the effect of a positive thinking skills training program on psychological adjustment among psychiatric patients’ caregivers.

**Method:**

A quasi-experimental research design (Non-Randomized Controlled Trial) was utilized in this study of one group (pre-, post-test, and follow-up after 3 months) in the psychiatric outpatient clinic at Port Said Psychiatric Health Hospital and Addiction Treatment. A purposive sample of 66 psychiatric patients’ caregivers. Eight sessions of positive thinking skills training were carried out in the form of small groups for four months. Two tools used for data collection consisted of Tool I: The Positive Thinking Scale, Tool II: The Psychological Adjustment Scale, in addition to the Personal and Clinical Data Sheet of the studied psychiatric patients’ caregivers.

**Results:**

The current study revealed that there were highly statistically ‎significant differences between the mean score of total psychological adjustment between pre-program and immediate post with mean difference ± Std. Error (23.51 ± 1.26) compared to the mean difference ± Std. Error in the pre-program and follow-up phase was (22.22 ± 1.20) While the mean difference ± Std. Error between the immediate post and follow-up phase was (1.29 ± 0.45) (*p* < 0.001) as determined by Cohen’s d test, there was a large effect of the training program on the immediate post and follow-up after 3 months.

**Conclusion:**

The positive thinking skill training program had a positive significant effect in improving the total psychological adjustment score among studied psychiatric patients’ caregivers. The study recommended the application of the positive thinking skill training program by the health profession at Port Said Psychiatric Health Hospital and Addiction Treatment to ensure continuity of care for psychiatric patients’ caregivers.

***Trial Registration Number (TRN)*:**

The study was approved by the Research Ethics Committee (REC), Faculty of Nursing/ Port Said University, Egypt (code number: NUR 13/3/2022–11). The study also retrospectively registered under the following number: PACTR202507513786427 on 1 July 2025.

**Supplementary Information:**

The online version contains supplementary material available at 10.1186/s12888-025-07276-3.

## Background

Psychiatric disorders are increasingly becoming a serious public health problem as the global prevalence of psychiatric disorders equivalent to be 10.7% of the global population, the studies estimated 9.3% for males and 11.9% for females [1]. Psychiatric illness has a negative impact on the functioning of the entire family because the family is seen as the primary caregiver and a pillar of support for mentally ill patients [2].

Long-term caring responsibility can drain the family’s energy, leaving them exhausted and more likely to experience negative emotions including feelings of hopelessness, guilt, depression, and helplessness. Evidence shows that family caregivers of mentally ill patients experience several difficulties and challenges that often lead to the caregiving burden. The term “caregiving burden” refers to physical, mental, emotional, and financial problems associated with caregiving [3]. A study reported that 73.6% of the family caregivers of individuals with mental disorders suffered from moderate to high levels of mental strain which resulted in a significant negative effect on different aspects of family caregivers’ lives, such as their quality of life, life expectancy, roles, and social interactions [4].

In Egypt, A study carried out at Assiut University Hospital showed that most study caregivers of psychiatric patients (92.5%) suffered from moderate to severe burden [5]. Another study conducted at Port Said Mental Health Hospital estimated that 83.1% of psychiatric patients’ caregivers have a severe level of burden and effect on their psychological well-being [6]. Psychological adjustment is crucial for caregivers to manage their caregiving burden, psychological adjustment can be defined as one’s subjective sense of distress and the degree to which one functions in daily life. Previous studies have indicated that a greater level of positive psychological adjustment is linked to better life satisfaction, quality of life, and lower levels of stress, anxiety, despair, and burnout. Additionally, people with high levels of psychological adjustment are typically more able to positively function in their daily lives [7]. A study conducted in India found that caregivers demonstrated inadequate psychological adjustment due to emotional strain, caregiver burden, lack of support, and financial hardship, which resulted in difficulty adapting to their caregiving roles [8].

Positive thinking is a cognitive process that helps individuals form optimistic ideas, make sound decisions, and increase their abilities to deal with challenging situations. On the other hand, it is a helpful strategy for coping with adverse situations and it has been linked to a higher quality of life and enhanced psychological and physical well-being of the caregivers [9]. By thinking positively, one can increase the ability to express attention to positive things and use positive language in interacting with others. It can change how a person views a problem and finds solutions. So, positive thinking can create a more positive environment, build self-confidence and solve problems creatively, increase productivity, success, and reduce stress [10].

The positive thinking skill training program specifically applied by the Psychiatric Mental Health Nurses (PMHN) due to their expertise in this field as they have a vital role toward psychiatric patients’ caregivers as they are in a good position to provide the necessary assistance and support to family caregivers to enable them to effectively cope with stressful situations by focusing on their needs. Mental health professionals should increase attention to the caregivers in addition to their patients and develop more programs for families; they should be provided social support, especially by healthcare professionals [11]. Hence, incorporating a positive thinking skill training program leads to enhancing the adoption of more effective coping styles by family caregivers to overcome their caregiver burden and adjust to stress [12].

## Theoretical framework

Cognitive Behavioral Theory (CBT), postulates that emotional and behavioral responses are largely shaped by individuals’ cognitive appraisals and interpretations of events. According to CBT, modifying dysfunctional or negative thought patterns through structured interventions such as cognitive restructuring, positive self-talk, and reframing can lead to improvements in emotional regulation and psychological well-being [13].

In the caregiving context, particularly for those supporting psychiatric patients, these individuals often experience significant emotional burden and psychological distress. CBT-based interventions aim to enhance coping skills, reduce stress, and promote adaptive emotional responses. Furthermore, recent developments in cognitive theory emphasize the psychological construction of emotions and cognition. This perspective views emotions as contextually shaped and cognitively assembled, supporting the use of CBT strategies to promote resilience and positive emotional experiences among caregivers [14].

Furthermore, the study also draws on Seligman’s Theory of Learned Optimism, which emphasizes that individuals can be taught to adopt more constructive explanatory styles, interpreting positive events as personal, permanent, and pervasive, while viewing negative events as external, temporary, and situation-specific. This optimistic explanatory style has been shown to reduce feelings of helplessness and promote psychological resilience. These theoretical foundations support the premise that positive thinking skills training can modify maladaptive cognitive patterns, and help the caregivers to develop more adaptive coping skills and improve their psychological adjustment [15].

### Significance of the study

Positive thinking skills are considered a unique strategy that focuses on increasing optimism, positive experiences, and positive affect as a means to mitigate stress, and depression and improve psychological adjustment [16]. People who think positively cope better with psychological stress, build great social support networks, and use more adaptive coping strategies [17]. An Egyptian study carried out to investigate positive thinking, resourcefulness skills, and future anxiety among the caregivers of children with intellectual disability in Zagazig City by Pourdavarani et al. [18] found the mean scores of positive thinking and total resourcefulness were higher at the post-intervention phase compared to pre-intervention among the participants’ caregivers. Otherwise, positive thinking improved among 60.76%, resourcefulness skills among 19.49%, and future anxiety decreased among 21.30% of the caregivers after the intervention, reported a significant improvement in their psychological state, alleviated their suffering, and having a positive impact on their care after positive thinking intervention.

Another study by Hadian, Fereidooni-Moghadam, and Maghsoodi [19], examined the effect of teaching positivity skills on life satisfaction among family members of patients with mental disorders, the study result indicated that the average score of life satisfaction increased in the intervention group immediately and one month after the intervention compared to before the intervention (*P* < 0.05). So, teaching positivity skills is recommended as a simple method to increase the life satisfaction of this group of patients.

Therefore, the present study was conducted to design and implement a positive thinking skills program among psychiatric patients’ caregivers to alleviate their suffering and improve their psychological state, as well as have a positive impact on their care to foster their psychological adjustment. The current study results reported a significant improvement in caregivers’ psychological adjustment scores after the program, suggesting that the positive thinking skill training program can be a valuable tool in mental health intervention. This study not only supports the efficacy of positive thinking skill training program but also provides a framework for future research and practical application in caregiver support programs.

### Study aim

This study aims to explore the effect of a positive thinking skills training program on psychological adjustment among psychiatric patients’ caregivers.

### Research hypothesis

The study hypothesized that there will be a statistically significant improvement in psychological adjustment among psychiatric patient’ caregivers after the implementation of the positive thinking skills training program.

## Methods

### Research design

A quasi-experimental study design was utilized to achieve the aim of this study [20]. Non-randomized comparative trial was used (comparison of one group at pre-, post-test, and after 3 months follow-up of the program) following the Guidelines for Reporting Non-Randomized Studies [21], and the Template for Intervention Description and Replication (TIDieR) checklist [22].

### Study setting

This study was conducted at the psychiatric outpatient clinic of Port Said Psychiatric Health Hospital and Addiction Treatment, located in Port Said Governorate, Egypt. The hospital is affiliated with the General Secretariat of Mental Health and Addiction Treatment (GSMHAT), Ministry of Health. The psychiatric outpatient clinic was accessible all days of the week from 10 AM to 2 PM.

### Study subjects

A purposive sample with a total number of 66 psychiatric patient’ caregivers who attended Port Said Psychiatric Health Hospital and Addiction Treatment were included in the study according to the inclusion criteria. A sample size of psychiatric patients’ caregivers was calculated according to the Open Epi-info version 7 programs using the following equation (23):

$$ {\text{Sample}}\,{\text{size}}({\text{n}}) = \frac{{{\text{N}} \times {\text{p }}\left( {{\text{1}} - {\text{p}}} \right)}}{{\{ ({\text{d}}^{{\text{2}}} /{\text{z}}^{{\text{2}}} _{{{\text{1}} - \alpha /2}} ) \times \left( {{\text{N}} - {\text{1}}} \right) + {\text{p }}\left( {{\text{1}} - {\text{p}}} \right)\} }} $$

- Population size (for finite population correction factor) (N) = 900 (The number of psychiatric patients’ caregivers who repeatedly visit the outpatient clinic over a period of three months at Port Said Psychiatric Health Hospital and Addiction Treatment).
- Expected frequency (p) = 50%, p= (0.5).
- Confidence limit (d) = 10%, d= (0.1).
- Confidence coefficient (z- score) = 90%, Z= (1.645).

So, the estimated sample size is 63, after adding the (5%) to avoid dropping out and/or incomplete responses or withdrawal, the final number for the sample size will be = 63 + 3 = **66** psychiatric patients’ caregivers.

### Inclusion criteria

- Being responsible for providing direct care to psychiatric patients for at least one year.
- Age (not less than 30 years).
- Agree to participate in the study.

### Tools for data collection

Two data collection instruments were used:

### Tool I - The Positive Thinking Scale (PTS)

The positive thinking scale was developed by Ayoub [24] in the Arabic language to assess individual positive thinking. It consists of 30 items and is divided into five subscales each subscale includes six items as follows: The first subscale is optimism and general satisfaction which includes items (1-6), the second subscale is emotional control and emotional intelligence from items (7-12), the third subscale is acceptance of the others and take responsibility from items (13-18), the fourth subscale is forgiveness and self-acceptance include items (19-24), finally the five subscale is a love of learning and taking risks present items from (25-30).

### Scoring system

The responses were measured on 3 points Likert scale; ranging from “1” to “3” including (never = 1, sometimes = 2, and always = 3). The score ranges from 30 (minimum score) to 90 (maximum score). The scores from 54 to 90 represent a higher degree of positive thinking suggesting that the individuals tend to be positive in viewing themselves, other people, or even the world. While a score from 30 to less than 54 represents a lower degree of positive thinking [25].

### Tool validity

The positive thinking scale was proven to be valid by Ayoub [24] as the tool was ascertained by a jury consisting of eleven experts in the field of psychology.

### Tool reliability

The positive thinking scale was tested for reliability by Ayoub [24] using Cronbach’s alpha coefficient (0.855), which indicates that the scale demonstrates acceptable internal consistency.

### Tool II - The Psychological Adjustment Scale (PAS)

The psychological adjustment scale was developed by Sari [26] in the Arabic language to assess individual adjustment to psychological stressors. The psychological adjustment scale consisted of 40 items and was divided into four subscales, the first subscale assesses personal adjustment which includes items from (1-9), and the second subscale assesses social adjustment from items (10-20). The third subscale assesses family adjustment from items (21-30), and the fourth subscale assesses emotional adjustment present items from (31-40). This scale used a two-point type scale “yes represent number (1) and “no” (0). The scale comprising 20 positive items and 20 negative items as follows:

Positive items: 1, 3, 6, 7, 8, 12, 15, 16, 18, 19, 22, 23, 25, 27, 29, 31, 32, 34, 35, 40.

Negative(reversed) items: 2, 4, 5, 9, 10, 11, 13, 14, 17, 20, 21, 24, 26, 28, 30, 33, 36, 37, 38, 39.

### Scoring system

If the total score is less than 17; it is counted as a low level of psychological adjustment. If the total score is between 17 and less than 34; it is counted as a moderate level of psychological adjustment. If the total score is 34 and above, it is counted as a high level of psychological adjustment [27].

### Tool validity

The psychological adjustment scale proved to be valid Fateel [27] as the content validity of the translated tools was ascertained by a panel of experts of three specialists in the field of psychology.

### Tool reliability

The psychological adjustment scale proved to be reliable Fateel [27] as Cronbach’s Alpha coefficient was (0.765) which indicates that the scale demonstrates a satisfactory internal consistency.

In addition, the Personal and Clinical Data Sheet; was developed in Arabic language after a review of the literature by the researcher and was revised by supervisors. The personal data of psychiatric patients’ caregivers includes (Age, gender, marital status, level of education, working status, residence, monthly income, number of family members, relationship between psychiatric patient & caregiver, and number of years for providing caregiving). As well as, clinical data; it includes: Medical health status, diagnosis of the psychiatric patient, and psychiatric family history.

### Pilot study

A pilot study was undertaken before starting the data collection phase. It was conducted from the first of October to the end of October 2022. The pilot study was carried out on 10% (seven caregivers) of the total sample of the studied caregivers of psychiatric patients who were selected randomly and met the inclusion criteria at Port Said Psychiatric Health Hospital and Addiction Treatment. The purpose of the pilot study was to test the applicability and feasibility of the positive thinking skill training program, and it served to estimate the time needed to complete the tools. It also helped to determine any obstacles and problems that might disrupt the data collection [28]. Studied caregivers who‎ shared in the pilot study ‎were ‎excluded from the entire sample of research work.

### Ethical considerations

The study was approved by the Research Ethics Committee (REC), Faculty of Nursing/ Port Said University/ Egypt with (code number: NUR 13/3/2022–11) based on the standard of the committee, Faculty of Nursing/ Port Said University and adhered to declaration of Helinski. The study also retrospectively registered under the following number: PACTR202507513786427 on 1 July 2025. All ethical issues were taken into consideration during all phases of the study and were included: Approval was taken from the General Secretariat of Mental Health and Addiction Treatment after an explanation of the study’s aim to conduct the study. In addition to this, written consent was taken from each participant (caregivers) after an explanation of the study aim and data collection process to be familiar with the importance of their participation. The studied caregivers were informed that their participation is voluntary and they have the right to withdraw from the study at any time without rationalization. Additionally, all data collected from the studied subjects were processed in total confidentiality and used only for the study. Code numbers instead of names of the participants were used for identification purposes. This measure ensured the participants would not be identified in the public reports.

### Fieldwork

Data collection was carried out at Port Said Psychiatric Health Hospital and Addiction Treatment from the beginning of November 2022 till the end of July 2023 for nine months. Data collection was conducted through four phases: Assessment phase, planning phase, implementation phase, and evaluation phase as the following:

*Phase I (Assessment Phase)*: The assessment phase was done from the first of November to the end of November 2022. The researcher obtained official permission to carry out the study visited the study settings, and arranged with the nursing director for the actual implementation of the study. The researcher met the psychiatric patients’ caregivers who satisfied the inclusion criteria and attended the outpatient clinic with their psychiatric patients either for receiving the treatment or regular follow-up at Port Said Psychiatric Health Hospital and Addiction Treatment two days/weekly (Monday and Tuesday). In this stage, the positive thinking skill training program was developed based on a comprehensive needs assessment for studied caregivers to identify specific areas where positive thinking could have the most significant impact. Non-randomized comparative trial was used (comparison of one group at pre-, post-, and after 3 months follow-up of the program) following the Guidelines for Reporting Non-Randomized Studies [17] and the Template for Intervention Description and Replication (TIDieR) checklist [18].

The orientation was done about the researcher’s name, the purpose of the study, the content of the study tools, how the program will be implemented, and finally the written formal consent to participate in the study. The researcher began to fill in the written pre-mentioned tools individually (pretest) in the form of Arabic language from psychiatric patients’ caregivers. The time needed for filling each one extended from 15 to 30 min depending on the response of each caregiver. As well as, the researcher obtained the telephone number of the caregiver to communicate with them about the training program, session time, and follow-up after the program implementation. The researchers also obtain information about the regular visits of the caregivers from hospital records with the assistance of a psychiatric nurse working at an outpatient clinic.

*Phase II (Planning Phase)*: Following the assessment phase results, the researchers constructed the positive thinking skill training program and session contents based on the identified caregivers’ needs and after review of the related literature. The identified needs, requirements, and knowledge limitations were translated into the training sessions’ goals and objectives. Furthermore, the researchers created a training booklet to assist caregivers in following the training sessions and serving as a reference at home, it was designed in simple Arabic language to be suitable for the caregiver’s level of understanding and supported by photos and illustrations to be distributed to caregivers at the beginning of the program implementation to help them to understand the program content. The program was structured to be interactive and included both individual and group exercises and a series of workshops and activities aimed at fostering positive thinking skills.

*Phase III (The session Implementation)*: The implementation phase is carried out from the beginning of December to the end of March 2023. The positive thinking skill training program was conducted in eight sessions two days/weekly (Monday and Tuesday) (Two sessions per day). The session varies in time from 30 to 55 min in addition to 10 min for open discussion and feedback from the caregivers. The sessions were conducted on a group basis which consisted of eight groups (seven groups composed of eight members and only one group composed of ten members) each group made a regular visit to the outpatient clinic once time every month so, the eight sessions finished after four months (two sessions per month for each group), the sessions built on each other to reinforce positive thinking habits as the following table.

**Table:** 

Week /Groups Months / Sessions	1st week		2nd week			3rd week		4th week		
	Monday	Tuesday	Monday		Tuesday	Monday	Tuesday	Monday		Tuesday
1st month / Session (1,2) (for all groups)	1st group (*n* = 8)	2nd group (*n* = 8)	3rd group (*n* = 8)		4th group (*n* = 8)	5th group (*n* = 8)	6th group (*n* = 8)	7th group (*n* = 8)		8th group (*n* = 10)
2nd month / Session (3,4) (for all groups)	1st group (*n* = 8)	2nd group (*n* = 8)	3rd group (*n* = 8)		4th group (*n* = 8)	5th group (*n* = 8)	6th group (*n* = 8)	7th group (*n* = 8)		8th group (*n* = 10)
3rd month / Session (5,6) (for all groups)	1st group (*n* = 8)	2nd group (*n* = 8)	3rd group (*n* = 8)		4th group (*n* = 8)	5th group (*n* = 8)	6th group (*n* = 8)	7th group (*n* = 8)		8th group (*n* = 10)
4th month / Session (7,8) (for all groups)	1st group (*n* = 8)	2nd group (*n* = 8)	3rd group (*n* = 8)		4th group (*n* = 8)	5th group (*n* = 8)	6th group (*n* = 8)	7th group (*n* = 8)		8th group (*n* = 10)

The sessions of the program were implemented using different teaching methods such as lectures, group discussion, brainstorming, role play, demonstration, re-demonstration of practical exercises, and homework assignment to reinforce and incorporate positive thinking skills in daily life as positive self-talk (I can, I’m strong), imagination to reduces anxiety, positive expectation for better access, affirm self-confidence for success, evoke positive thoughts for motivation, avoid comparison with other people, identify strengths and weakness points, overcome weakness, and look at things rationally and use problem-solving skills. In addition, different audiovisual materials were used as PowerPoint, pictures, videos, and handouts to facilitate the teaching of each topic. The details regarding the training program sessions shifted to “Supplementary Material”.

Moreover, the booklet was printed and given to each caregiver as an incentive to attract attention, motivate, and help to review it at home, as well as giving recognition and reward with small tokens of appreciation and simple gifts (sweet, light snacks) offered to participants to encourage completing the sessions, additionally after completing the program more facilitation was provided to the participant by help them to access to follow up support sessions if they need to ensure continue of applying the positive skills in their daily lives as the researcher telephone number was available for all study participant. The researcher emphasized that this session was done for teaching purposes not for evaluation, so mistakes and forgetting were allowed and were corrected immediately by the researcher. The researcher evaluates the effectiveness of the program in the last session (session eight) using the previously mentioned tools (post-test).

*Phase IV (Evaluation phase)*: The effectiveness of the positive thinking skill training program on the psychological adjustment of the psychiatric patients’ caregivers evaluated immediately after the implementation of the program (post-test) through the pre-mentioned tools and comparing the pre-test with the post-test. This process was carried out again after three months (follow-up test) (from the 1st of July to the end of July 2023). Finally, the studied caregivers were thanked for the effort and the time they offered.

### Statistical design

The collected data was organized, revised, stored, tabulated, and analyzed using SPSS for Windows version 20.0 (SPSS, Chicago, IL). Continuous data were normally distributed and are expressed as the mean ± standard deviation (SD). Categorical data are expressed numbers and percentages. Data was presented using suitable tables and figures. The chi-square test (X^2^) was used for the comparison of variables with categorical data. Pearson’s correlation coefficient was used to test the correlation between variables and a t-test, ANOVA was used to compare the means. Multiple linear regression (step-wise) was also employed to predict factors influencing the positive thinking degrees or psychological adjustment levels. A significant level value was considered when the p-value ≤ 0.05 and highly statistically significant at *P* value ≤ 0.01. Cohen’s d used to measure the effect size was considered small effect size at < 0.5, medium effect size at 0.5 < 0.8, and large effect size at > 0.8.

## Results

### Personal characteristics of psychiatric patients’ caregivers

Table 1 Reveals the personal characteristics of the studied psychiatric patients’ caregivers, as shown, more than two-thirds of them (68.2%) were female. As regards the caregivers’ age, their age ranges between 30 and 63 years old with mean ± SD of 47.12 ± 8.58, less than half of them (45.5%) were aged from 40 to less than 50 years old. According to their residence, less than three-quarters of the psychiatric patients’ caregivers (71.2%) lived in urban. The table also shows that 65.2% of the studied caregivers were working and 78.8% reported that their monthly income was insufficient.

**Table 1:** Personal characteristics of the psychiatric patients’ caregivers

Personal Characteristics		Psychiatric patients’ caregivers *n* = 66		
		No.		%
Gender				
Male Female		21 45		31.8 68.2
Age/ years				
30 - <40 40 - <50 50 - < 60 ≥ 60		12 30 17 7		18.2 45.4 25.8 10.6
Range Mean ± SD		30–63 47.12 ± 8.58		
Marital status				
Single Married Widow Divorced		7 27 14 18		10.6 40.9 21.2 27.3
Educational levels				
Not read and write Basic education Secondary University		6 10 27 23		9.1 15.2 40.9 34.8
Residence				
Urban Rural		47 19		71.2 28.8
Working status				
Working Not working		43 23		65.2 34.8
Monthly income				
Sufficient Insufficient		14 52		21.2 78.8
Number of family members				
3–4 5 or more		38 28		57.5 42.5
Range Mean ± SD		3–8 4.51 ± 1.05		
Relation between caregiver and psychiatric patient:				
Father Mother Brother/sister Husband/wife		14 40 7 5		21.2 60.6 10.6 7.6
Duration for providing caregiving/years:				
2-<5 5 or more		12 54		18.2 81.8
Range Mean ± SD		2–8 5.06 ± 1.65		

The table also illustrates that less than two-thirds of them (60.6%) their mothers consider the primary direct caregiver provider. In addition, 81.8% of them provide caregiving for 5 years or more with a mean ± SD of 5.06 ± 1.65 years.

### Clinical characteristics of psychiatric patients’ caregivers

Table 2 Presents the distribution of the psychiatric patients’ caregivers according to clinical characteristics, the results revealed that more than half of them (51.5%) suffered from chronic medical conditions. Looking at their psychiatric diagnosis 56.1% of the studied caregivers had their patient diagnosed with schizophrenia. The table also clarifies that 43.9% of the studied caregivers had a psychiatric family history and more than three-quarters of them (79.3%) were second-degree relatives.

**Table 2:** Clinical characteristics among the psychiatric patients’ caregivers

Clinical characteristics	Psychiatric patients’ caregivers *n* = 66	
	No.	%
Chronic medical condition		
Yes No	34 32	51.5 48.5
Psychiatric diagnosis:		
Schizophrenia Mania Depression	37 18 11	56.1 27.3 16.6
Psychiatric family history:		
Yes No	29 37	43.9 56.1
Psychiatric family relation: N = 29		
First degree relative Second degree relative	6 23	20.7 79.3

### Total positive thinking degrees among the psychiatric patients’ caregivers in pre, immediate post, and follow-up of the program

**Fig. 1:**
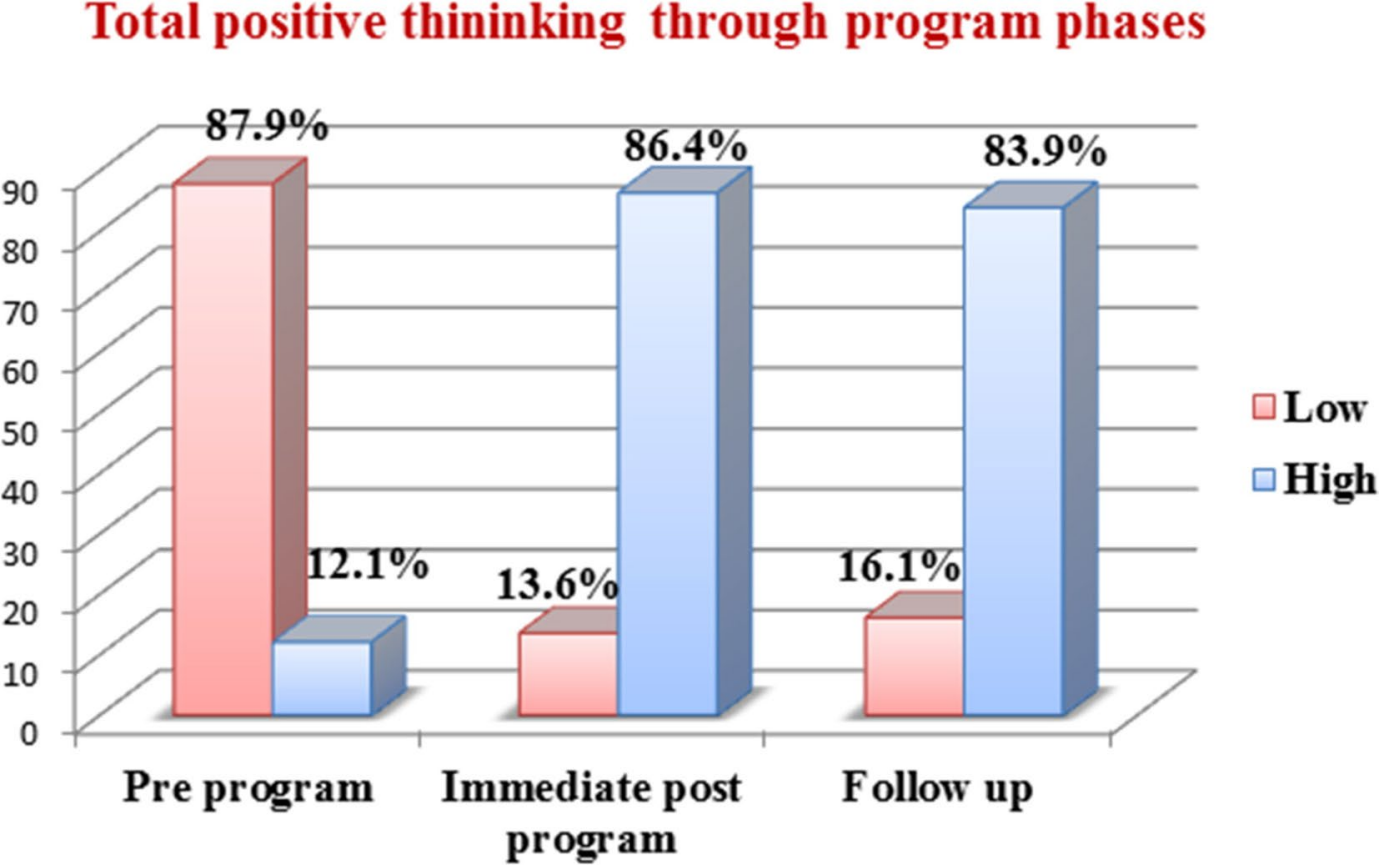
Total positive thinking degrees among the psychiatric patients’ caregivers in the pre-, immediate post, and after 3 months follow-up of the program

Figure 1 shows improvements in total positive thinking scores among the psychiatric patients’ caregivers throughout the program phases, the figure implied that the minority of the psychiatric patients’ caregivers (12.1%) had a higher degree of positive thinking in the pre-program phase and the percentage improved to 86.4% at immediate post-program phase and declined to 83.9% at follow up phase.

### Comparison of the mean difference of positive thinking score and its subscale among the psychiatric patients’ caregivers at the pre-, immediate post, and score after 3 months follow-up of the program

Table 3 Compare the mean difference of positive thinking score and its subscale among the psychiatric patients’ caregivers at the pre-, immediate post, and after 3 months follow-up of the program implementation. The table shows that there was a highly statistically significant improvement in immediate post-program and at follow-up phase after 3 months compared with pre-program among the studied caregivers in all subscale (*p* < 0.001) as determined by Cohen’s d test, the effect size of the training program is large on the total positive thinking scores immediately post-program and at follow up phase (Cohen’s d = 2.8793, 2.7336 respectively).

**Table 3:** Comparison of the mean difference of positive thinking score and its subscale among the psychiatric patients’ caregivers at the pre-, immediate post, and score after 3 months follow-up of the program

Positive thinking subscale		Repeated measures ANCOVA					Pairwise Comparisons					
		Sum of Squares	df	Mean Square	Eta Square	F/*p*-value	Pre& Immediate post		Pre& follow up (after 3 months)		Post & Follow-up (after 3 months)	
							Mean Difference ± Std. Error	t/*p*-value	Mean Difference ± Std. Error	t/*p*-value	Mean Difference ± Std. Error	t/*p*-value
Optimism and general satisfaction	Time	29.06	1	29.06	0.903	F=15.22 *P*<0.001**	6.452±0.277	t=23.29 *P*<0.001**	6.387±0.331	t=19.29 *P*<0.001**	0.065±0.264	t=0.246 *P*=1.000
	Error	1.68	61									
Emotional control and emotional intelligence	Time	25.07	1	25.07	0.987	F=15.46 P<0.001**	6.823±0.399	t=16.96 *P*<0.001**	6.855±0.404	t=16.96 *P*<0.001**	0.032± 0.223	t=0.143 *P*=1.000
	Error	1.62	61									
Acceptance of others and taking responsibility	For time	22.77	1	22.77	0.986	F=13.24 *P*<0.001**	7.323±0.378	t=19.37 *P*<0.001**	7.194±0.406	t=17.71 *P*<0.001**	0.129±0.219	t=1 *P*=1.000
	Error	1.71	61									
Forgiveness and self-acceptance	Time	35.22	1	35.22	0.987	F=22.92 *P*<0.001**	5.839±0.400	t=14.59 *P*<0.001**	5.887±0.406	t=14.50 *P*<0.001**	0.048±0.233	t=0.206 *P*=1.000
	Error	1.53	61									
Love of learning and taking risks	Time	20.93	1	20.93	0.982	F=8.81 *P*<0.001**	7.419±0.367	t=20.11 *P*<0.001**	6.968±0.396	t=17.59 *P*<0.001**	0.452±0.256	t=1.76 *P*=0.247
	Error	2.55	61									
The total score of positive thinking	Time	26.54	1	26.54	0.989	F=11.86 *P*<0.001**	34.24±1.53	t=22.37 *P*<0.001**	33.74±1.56	t=21.62 *P*<0.001**	0.500±0.67	t=0.746 P=1.000
	Error	2.24	61									
	Cohen’s d value						2.8793		2.7336		0.1944	

Cohen’s d: small effect size at < 0.5, medium effect size at 0.5 < 0.8, large effect size at > 0.8, t-pairwise comparison (*) Statistically significant at *P* 0.05 (**) Highly statistical significance at *P* < 0.01; comparisons were adjustment by using Bonferroni correction

### Total psychological adjustment levels among the psychiatric patients’ caregivers in pre, immediate post, and follow-up of the program

**Fig. 2:**
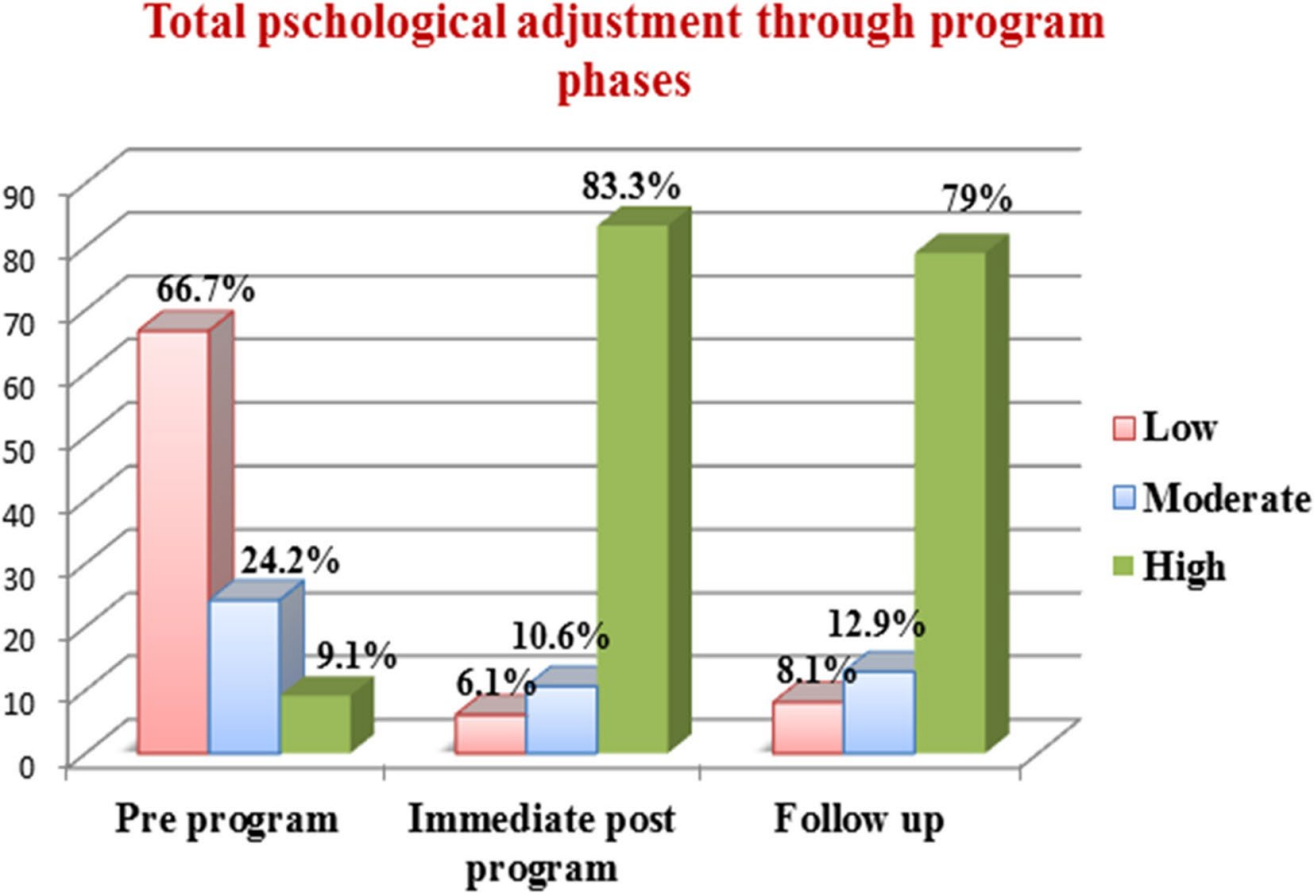
Total psychological adjustment levels among the psychiatric patients’ caregivers in the pre-, immediate post, after 3 months follow-up of the program

Figure 2 presents improvements in total psychological adjustment score among the psychiatric patients’ caregivers throughout the program phases, the figure reveals that the minority of them (9.1%) had a high level of psychological adjustment in the pre-program phase and the percentage increased to 83.3% at immediate post-program phase and declined to 79% at follow up phase.

### Comparison of the mean difference of psychological adjustment score and its subscale among the psychiatric patients’ caregivers at pre-, immediate post, and after 3 months follow-up of the program

Table 4 Compare the mean difference of psychological adjustment score and its subscale among the psychiatric patients’ caregivers at pre-, immediate post, and after 3 months follow-up of the program implementation. The table illustrates that there was a highly statistically significant improvement in immediate post-program and after 3 months follow-up of the program compared with pre-program among the studied caregivers in all subscale (*p* < 0.001) as determined by Cohen’s d test, the effect size of the training program is large on the total psychological adjustment scores immediately post-program and at follow up phase (Cohen’s d = 2.4279, 2.3364respectively).

**Table 4:** Comparison of the mean difference of psychological adjustment score and its subscale among the psychiatric patients’ caregivers at pre-, immediate post, and after 3 months follow-up of the program

Psychological adjustment subscale		Repeated measures ANCOVA					Pairwise Comparisons					
		Sum of Squares	df	Mean Square	Eta square	F/*p*-value	Pre& Immediate post		Pre& follow up (after 3 months)		Post & Follow-up (after 3 months)	
							Mean Difference ± Std. Error	t/*p*-value	Mean Difference ± Std. Error	t/*p*-value	Mean Difference ± Std. Error	t/*p*-value
Personal adjustment	Time	6.74	1	6.74	0.970	F=7.23 *P*<0.001**	5.694±0.342	t=16.64 *P*<0.001**	5.565±0.335	t=16.61 *P*<0.001**	0.129±0.171	t=0.754 *P*=1.000
	Error	0.93	61									
Social adjustment	Time	9.88	1	9.88	0.955	F=6.99 *P*<0.001**	6.242±0.405	t=15.41 *P*<0.001**	5.855±0.432	t=13.55 *P*<0.001**	0.387± 0.230	t=1.68 *P*=0.294
	Error	1.41	61									
Family adjustment	Time	13.84	1	13.84	0.962	F=14.23 *P*<0.001**	5.016±0.440	t=11.40 *P*<0.001**	4.661±0.450	t=10.35 *P*<0.001**	0.355±0.193	t=1.83 *P*=0.211
	Error	0.97	61									
Emotional adjustment	Time	7.41	1	7.41	0.965	F=6.88 *P*<0.001**	6.113±0.372	t=16.43 *P*<0.001**	5.935±0.381	t=15.57 *P*<0.001**	0.177±0.235	t=0.753 *P*=1.000
	Error	1.07	61									
Total psychological adjustment score	Time	8.76	1	8.76	0.968	F=5.43 *P*<0.001**	23.516±1.268	t=18.54 *P*<0.001**	22.226±1.209	t=18.38 *P*<0.001**	1.290±0.453	t=2.84 *P*=0.018*
	Error	1.61	61									
	Cohen’s d value						2.4279		2.3354		0.36204	

Cohen’s d: small effect size at < 0.5, medium effect size at 0.5 < 0.8, large effect size at > 0.8, t: pairwise comparisons (*) statistically significant at *P* < 0.05 (**) highly statistical significance at *P* < 0.01; comparisons were adjustment by using bonferroni correction

### Relation between positive thinking and psychological adjustment among the psychiatric patients’ caregivers throughout the program phases

Table 5 Elaborates that there was a statistically significant relation between positive thinking and psychological adjustment among the psychiatric patients’ caregivers in the pre-program, immediate post-program, and follow-up program phase (*P* < 0.001).

**Table 5:** Relation between positive thinking and psychological adjustment among the psychiatric patients’ caregivers throughout the program phases

Psychological adjustment levels	Positive thinking degrees											
	Pre-program (*n* = 66)				Immediate post-program (*n* = 66)				Follow-up program (after 3 months) (*n* = 62)			
	Lower degree (*n* = 58)		Higher degree (*n* = 8)		Lower degree (*n* = 9)		Higher degree (*n* = 57)		Lower degree (*n* = 10)		Higher degree (*n* = 52)	
	No	%	No	%	No	%	No	%	No	%	No	%
Low psychological adjustment	44	75.9%	0	0.0	4	44.4%	0	0.0	5	50%	0	0.0
Moderate psychological adjustment	14	24.1%	2	25%	4	44.4	3	5.3	2	20%	6	11.5
High psychological adjustment	0	0.0	6	75%	1	11.2	54	94.7	3	30%	46	88.5
Test of significance (χ2, *P*)	χ^2^= 49.571, *P* < 0.001**				χ^2^ = 43.107, *P* < 0.001**				χ^2^ = 30.092, *P* < 0.001**			

χ^2^: Chi square (*) Statistically significant at *P* < 0.05 (**) Highly statistical significance at *P* < 0.001

### Relation between socio-demographic factors with the positive thinking degrees and psychological adjustment levels

#### Stepwise multiple linear regression for predicting factors that affect positive thinking degrees

Table 6 Indicates that the caregivers’ age (40 - <50) and education level (university education), were a statistically significant positive predictor of the positive thinking score (*p* = 0.038, 0.000 respectively). The model explains 51% of the variation in positive thinking, as shown by the value of r-square. Other caregivers’ characteristics did not influence positive thinking scores.

**Table 6:** Stepwise multiple linear regression for predicting factors that affect positive thinking degrees

Model	Unstandardized coefficients		Standardized Coefficients	T	*P*. value	95.0% Confidence interval for B	
	B	Std. Error	Beta			Lower bound	Upper bound
Constant	68.031	6.131		11.096	0.000**	55.775	80.286
Age (40 - <50)	4.582	2.160	0.211	2.122	0.038*	0.265	8.900
Educational levels (University education)	3.650	0.790	0.473	4.623	0.000**	2.072	5.229

(a) Dependent variables: Total positive thinking score. (b) Predictors: (Constant) Variables entered and excluded: Caregiver gender, age, marital status, education level, residence, working status, monthly income, number of family members, relation between caregiver and psychiatric patient, duration for providing caregiving, chronic medical condition of the caregiver, psychiatric diagnosis, psychiatric family history, psychiatric family relation

* Statistically highly significant (*p* < 0.05). ** Statistically highly significant (*p*< 0.001). R-square = 0 0.511, ANOVA: F = 3.478, *p* < 0.001

#### Stepwise multiple linear regression for predicting factors that affect psychological adjustment level

Table 7 Illuminates that the age of psychiatric patients’ caregivers (40–<50), gender (female), education level (university education), number of family members, and duration of caregiving (5years or more) was a statistically significant positive predictor for high psychological adjustment score (*p* = 0.045, 0.030, 0.002, 0.014, 0.026 respectively). While, the insufficient income, was a statistically significant positive predictor for low level of psychological adjustment (*p* = 0.034). The model explains 37% of the variation in psychological adjustment, as shown by the value of the r-square. Other caregiver’s characteristics did not influence the psychological adjustment score.

**Table 7:** Stepwise multiple linear regression for predicting factors that affect psychological adjustment level

Model	Unstandardized Coefficients		Standardized Coefficients	T	*P*. value	95.0% Confidence interval for B	
	B	Std. Error	Beta			Lower bound	Upper bound
Constant	3.614	0.505		7.160	0.000*	1.678	3.097
Age (40 - <50)	0.205	0.113	0.332	1.819	0.045*	0.021	0.431
Gender (female)	0.494	0.221	0.423	2.238	0.030*	0.051	1.938
Educational levels (University education)	0.155	0.048	0.373	2.215	0.002**	0.059	0.251
Income (insufficient)	– 0.556	0.255	– 0.510	– 2.184	0.034*	– 0.045	– 1.067
Number of family members (3:4)	4.793	2.100	0.234	2.282	0.026*	8.991	0.594
Duration of caregiving (5 years or more)	0.437	0.172	0.397	2.540	0.014*	0.783	0.091

(a) Dependent variables: Total psychological adjustment score. (b) Predictors: (Constant) Variables entered and excluded: Caregiver gender, age, marital status, education level, residence, working status, monthly income, number of family members, relation between caregiver and psychiatric patient, duration for providing caregiving, chronic medical condition of the caregiver, psychiatric diagnosis, psychiatric family history, psychiatric family relation, total positive thinking score

* Statistically highly significant (*p* < 0.05). ** Statistically highly significant (*p* < 0.001). R-square = 0.375, ANOVA: F = 10.337, *p* < 0.001.

## Discussion

Globally, over 450 million individuals suffer from mental disorders worldwide with a projected increase by 2030 mostly in developing countries [29]. Although families are considered a vital source of support, the related responsibilities of care often impose significant stress that impacts the physical, emotional, psychological, social, financial, and mental well-being of caregivers and potentially all family members. The study showed that family caregivers of close relatives with mental illnesses often experience distress, anxiety, and depression and face economic problems that require significant support and training to enhance the proper adjustment and reduce their caregiver burden [30].

Incorporating a positive thinking skill training program for family caregivers of psychiatric patients is intended to help the individuals to identify their positive experience to coping with stress and strengthen their self-respect and self-esteem, well-being, reducing their rumination, and establishing peace of mind, hope, and optimal functioning and facilitate changes in individuals behavior [31]. So, this study was conducted to explore the effect of a positive thinking skills training programs on psychological adjustment among psychiatric patients’ caregivers.

The finding of the present study denoted that there was a significant improvement in total positive thinking degrees at the immediate post and after 3 months follow-up of the program compared with the pre-program among studied psychiatric patients’ caregivers. This result may be due to a lack of training programs provided to psychiatric patients’ caregivers about ways to think positively. So, the positive thinking skill training effectively works to increase the caregivers’ positive personal impressions resulting in accepting their responsibility for care, so the caregivers learn to focus on positive aspects and maintain a hopeful outlook, which enhances their positive thinking skills.

The results are in the same context as Taherkhani et al. [25] who conducted a randomized controlled trial at Shiraz University of Medical Sciences in Iran and found that the positive thinking intervention significantly increased the mean scores of positive thinking at baseline one week and two months after the training. The current results are also incongruence with a study carried out in India to explore the impact of positive cognitive behavior therapy-based intervention on a caregiver of a patient with mental illness, the result concluded that the caregiver reported a remarkable decrease in caregiver strain, depression, anxiety, greater satisfaction with life and a high score of total positive thinking after implementation the intervention [32].

Also, a study conducted in Iran clarified that after the implementation of a group psychological training program for family caregivers of patients with schizophrenia, a satisfactory level of the total positive thinking scores increased from nearly one-third in the pre-test to most of them in the post-test and recommended that, implementing educational training courses in future studies with a greater sample size by health service providers can improve the continuity of care among caregivers of psychiatric patients [33].

Additionally, the findings of the current study clarified that there were highly statistically significant differences between the mean scores of five subscales of positive thinking concerning optimism and general satisfaction, emotional control and emotional intelligence, acceptance of others and taking responsibility, forgiveness and self-acceptance and love of learning and taking risks in pre, immediate post, and follow-up phase of the program. These study findings may be attributed to positive thinking affecting the caregivers’ awareness about how to cope with their life challenges, enhancing the use of different strategic methods, and acquiring a more optimistic view after implementation of the positive thinking skills training.

These results are congruent a study conducted in Iran by Haroon and Bahiraei [34] showed that there was a significant difference between experimental and control groups in terms of life satisfaction after positivism training. In the same vein, the result matched with the study in Indonesia mentioned that the self-acceptance score increased after they received positive thinking training among the experimental group and led to improvement in overall psychological well-being [35]. Also, the results were aligned with an Egyptian study performed by Zoromba et al. [36] which demonstrated that there was a significant improvement in the scores of well-being, self-control, emotionality, sociability, and total emotional intelligence scores after implementation of emotional intelligence training program.

Further, the present study revealed that there was a significant improvement in total psychological adjustment levels at the immediate post and after 3 months follow-up of the program compared with the pre-program among studied psychiatric patients’ caregivers. This could related to the structure and the content of the program worked on supporting the family caregivers to understand and accept their patients’ illness, and caregiving role, improving their ability to make proper decisions, adapting to the stress and challenges in different areas of daily living. Consequently, this results in an improvement in their total psychological adjustment.

The results are approved with a study conducted in Egypt by El-Azzab, Ali, and Ahmed [37] who mentioned that the mean score of psychological adjustment improved post-program nursing intervention. This result is also similar to a study conducted in China found that more than two-fifths of the studied caregivers had high total psychological adaptation with their psychiatric patient’s condition obtained from the measurements before, after, and three and six months after the psycho-education intervention application [38]. Similarly, a quasi-experimental study in Iran concluded that a better psychological adaptation to stressful situations and increased happiness after positive thinking training intervention [39]. So, a study carried in India by Pathak and Mathew [8], suggested that psychological support and guidance strategies play a crucial role in improving the psychological well-being among the caregiver of persons with mental illness and enhance their coping skills and emotional stability.

The current study showed that there were highly statistically significant differences between the mean score of four subscales of psychological adjustment regarding personal, social, family, and emotional subscales and the mean score of total psychological adjustment in pre, post, and follow-up of the program. This finding reflects the effectiveness of a positive thinking training program in enhancing all aspects of psychological adjustment among psychiatric patients’ caregivers. These study findings are consistent with Jiang et al. [38] who revealed that there was a significant difference between caregivers’ social, psychological, and self-care adaptation after professional support provided to them in the training program. In the same direction a quasi-experimental study conducted in Turkey where clarified that most of the studied caregivers of psychiatric patients reported good psychological adaptation after a psycho-education program based on the family intervention model compared to the minority of them in pre-program [40].

The finding of the present study demonstrated that older adult caregivers were a positive predictor factor for higher positive thinking skills, The possible explanation is that older age caregivers are more skillful and highly experienced in dealing positively with everyday life events, so the caregivers under the age 30 year were excluded from the present study as they are less experienced to apply adaptive coping strategies compared to an older adult, as well as the younger caregivers usually are busy due to early career responsibilities and deal with unique different life challenges. The result agrees with Taherkhani et al. [25] confirmed that positive thinking interventions can increase older adults’ resilience and improve their quality of life which leads to greater life satisfaction.

University-educated caregivers are considered another predictor factor for high positive thinking skills and high psychological adjustment levels. It may be due to that the high educational level plays an important role in helping psychiatric patients’ caregivers acquire different knowledge and experience, increase their understanding and access to resources and support that can help them deal with stress and burden related to the care of their relatives and leads to adopting positive coping strategies to face the difficulties and challenges regarding their patients’ responsibility. This finding is in the same direction as Almeida and Ifrim [41] who confirmed that less than two-thirds of the studied sample who were a higher education including Bachelor’s, Master’s, and Doctorate degrees reported a higher score of positive thinking skills. Similarly, a study performed in Egypt mentioned that the higher-educated caregivers reported high positive thinking skills post-implementation of the program [42].

The result is in the same direction as a study in Tanzania by Clari et al. [43] who found that there were statistically significant relations between the caregivers’ education and their total psychological adjustment in post-program and suggested that caregivers with lower education tended to use of maladaptive coping skills and present more anxiety, depression, more psychological distress. Also, a study in China conducted by Zhou et al. [44] to investigate the associations between caregiving knowledge and skills with caregiver burden, psychological well-being, and coping styles among family caregivers of people living with schizophrenic patients, confirmed that a high percentage of family caregivers with a higher level of knowledge, experiences, and skills were commonly among university-educated caregivers who reported more psychological well-being, and high positive coping styles in all aspects of daily life.

Insufficient income and an increased number of family members were predicted a lower psychological adjustment level. It may be due to the fact that low income is a burden on caregivers and makes them live all the time with worries, uncertainty, and doubts about their ability to provide care and satisfy the needs of their psychiatric patients from one side and maintain a standard of living which contributes to feelings of security, life satisfaction from the other side. At the same time, an increase in the number of families leads to a greater physical and financial burden, so the caregivers are afraid regarding their future life needs, education, and employment.

The results are supported by an Egyptian study carried out by Sebaie et al. [39] to evaluate the effect of the intervention program on positive thinking, resourcefulness skills, and future anxiety among the caregivers of children with intellectual disability which revealed that the future anxiety and psychological distress was high among insufficient-income caregivers and those who had more than three children in their family. In the same line, a study conducted in India among caregivers of persons with mental illness reported that less than half of the studied sample experienced high levels of psychological distress, and burden particularly among those with insufficient income [8].

Another positive predictor factor of a high psychological adjustment level was the increased duration of providing caregiving. This may be due to the fact over time, the caregivers acquired more skills and experiences such as developing better coping mechanisms and problem-solving skills. This increased resourcefulness helps them manage stress more effectively linked to higher levels of psychological well-being. These results are in line with Fannick [45] in the USA found that an increased length of time as a caregiver was associated with lower caregiver burden, high physical, and mental health, as well as increased psychological resilience among caregivers of patients living with dementia. Moreover, the study in the USA discovered that the long-term caregivers who provided an average of 7.69 years of care were able to find meaning in their role compared to short-term caregivers and the study also reported that the longer duration of care tended to be associated with higher positive aspects of caregiving and development of psychological resilience and positive adaptation, higher quality of life and higher motivation in the caregiving role, additionally adjusted for perceived stress and caregiving strain [46].

## Conclusion

### Based on the findings of the present study, it can be concluded that

The positive thinking skill training program had a positive significant effect in improving the total psychological adjustment score among studied psychiatric patients’ caregivers.

### Implications

Providing ongoing support, adequate guidance, training, and resources for psychiatric patients’ caregivers such as support groups or counseling services, can help them maintain a positive mindset and develop constructive thinking skills to cope with their challenges at Port Said Psychiatric Health Hospital and Addiction Treatment. Application of the positive thinking skill training program by the health profession at Port Said Psychiatric Health Hospital and Addiction Treatment to ensure continuity of care to psychiatric patients’ caregivers.

### Limitation of the study

There are certain limitations to this study. The sample size must first be increased. Second, it was not possible to use randomization or a control group in this study, and this issue can be affected by personal bias. The third limitation is the absence of follow-up actions to ensure that the outcomes are stable. Additionally, In the follow-up phase, four caregivers were missed as the doctor referred them to the inpatient department due to the worsening of the symptoms of their patients outside Port Said Psychiatric Health Hospital and Addiction Treatment because all wards were completed. Finally, the researcher took more time to collect data from the psychiatric patients’ caregivers and apply the positive thinking training session due to that the psychiatric patients’ caregivers have a regular visit only every month. To address this issue, allow the participation of the psychiatric patients’ caregivers to observe the effect of the positive thinking training program on their psychological adjustment in daily life to overcome the concern of bias. Further research should be done on a larger sample of psychiatric patients’ caregivers in various settings to generalize the findings.

## Supplementary Information

Below is the link to the electronic supplementary material.

Supplementary Material 1

## Data Availability

Due to confidentiality concerns, the data and materials used in the current study cannot be made publicly available. However, they are available from the corresponding author upon reasonable request.

## Abbreviations

Abbreviations: Meaning
CBT: Cognitive Behavioral Theory
GSMHAT: General Secretariat of Mental Health and Addiction Treatment
PMHN: Psychiatric Mental Health Nurse
PTS: Positive Thinking Scale
PAS: Psychological Adjustment Scale

## Declarations

### Clinical trail number

The study was retrospectively registered under the following number: PACTR202507513786427 on 1 July 2025.

## References

[1] Dattani S, Ritchie H, Roser M. (2021). *Mental health*, Published online at Our World In Data. Org. Retrieved from: https://ourworldindata.org/mental-health

[2] Rahmani F, Roshangar F, Gholizadeh L, Asghari E. Caregiver burden and the associated factors in the family caregivers of patients with schizophrenia. Nurs Open. 2022;9(4):1995–2002. 10.1002/nop2.1205. 35347867 10.1002/nop2.1205 PMC9190687

[3] James R, Tungol J. Aggravating factors and adverse impacts of the subjective burden of family caregivers of schizophrenia patients. Indian J Health Wellbeing. 2021;12(4):413–6. https://iahrw.org/our-services/journals/indian-journal-of-health-wellbeing/.

[4] Bagheriamiri Z, Mirsepassi Z, Sayadi L. Caregiver burden, attachment, and cognitive emotion among the family caregivers of severe mental illness patients. BMC Psychol. 2024;12(1):610. 10.1186/s40359-024-02111. 39482790 10.1186/s40359-024-02111-y PMC11529072

[5] Ebrahim O, Al-Attar G, Gabra R, Osman D. Stigma and burden of mental illness and their correlates among family caregivers of mentally ill patients. J Egypt Public Health Assoc. 2020;95(1):1–9. 10.1186/s42506-020-00059-6. 33164132 10.1186/s42506-020-00059-6 PMC7649189

[6] Goda S, Megahed M, Hamza H. Relation between the burden of caregiving, submissive behaviors, and depressive symptoms among caregivers of psychiatric patients. Int Egypt J Nurs Sci Res. 2022;3(1):240–58. 10.21608/EJNSR.2022.247077.

[7] Arslan G, Yıldırım M, Zangeneh M. Coronavirus anxiety and psychological adjustment in college students: exploring the role of college belongingness and social media addiction. Int J Mental Health Addict. 2022;20(6):1–14. 10.1007/s11469-020-00460-4. 10.1007/s11469-020-00460-4 PMC7819624 33500688

[8] Pathak A, Mathew K. Need for care to caregivers: psychological distress and its socio-demographic correlates among the relatives of persons with mental illness. Int J Psychosoc Rehabil 2017;21(2):3–12. 10.61841/7c09ch76.

[9] Bekhet A, Garnier-Villarreal M. Effects of positive thinking on caregivers’ burden and care-recipients’ behavioral problems. West J Nurs Res. 2020;42(5):365–72. 10.1177/0193945919861970. 31267839 10.1177/0193945919861970

[10] Na’imah T, Dwiyanti R, Sriyanto S, Ismail F. Development of a positive thinking measuring tool for young Indonesian Muslims. Int J Islamic Educ Psychol. 2023;4(1):17–32. 10.18196/ijiep.v4i1.17869.

[11] Noori L, Ebrahim S. Family caregivers burden and coping strategies for patients with mentally ill patient in Mosul City. Mosul J Nurs. 2020;8(2):215–24. 10.33899/mjn.2020.167118.

[12] Søvold L, Naslund J, Kousoulis A, Saxena S, Qoronfleh M, Grobler C, Münter L. Prioritizing the mental health and well-being of healthcare workers: an urgent global public health priority. Front Public Health. 2021;9:679397. 10.3389/fpubh.2021.679397. 34026720 10.3389/fpubh.2021.679397 PMC8137852

[13] Beck J. Cognitive behavior therapy: basics and beyond. 2nd ed. New York: Guilford Press; 2011.

[14] Tiba A. Psychological construction as a theoretical principle for guiding cognitivebehavioral treatments. Front Psychol. 2024;15:1363819. 10.3389/fpsyg.2024.1363819. 38566941 10.3389/fpsyg.2024.1363819 PMC10985133

[15] Seligman M, Steen T, Park N, Peterson C. Positive psychology progress: empirical validation of interventions. Am Psychol. 2021;76(5):543–56. 10.1037/amp0000700. 10.1037/0003-066X.60.5.410 16045394

[16] Griva K, Chia J, Goh Z, Wong Y, Loei J, Thach T, Khan B. Effectiveness of a brief positive skills intervention to improve psychological adjustment in patients with end-stage kidney disease newly initiated on hemodialysis: protocol for a randomized controlled trial (HED-Start). BMJ Open. 2021;11(9):53–88. 10.1136/bmjopen-2021-053588. 10.1136/bmjopen-2021-053588 PMC8458344 34548369

[17] Sabouri F, Rambod M, Khademian Z. The effect of positive thinking training on hope and adherence to treatment in Hemodialysis patients: a randomized controlled trial. BMC Psychol. 2023;11(1):1–8. 10.1186/s40359-023-01036-2. 36624540 10.1186/s40359-023-01036-2 PMC9830796

[18] Pourdavarani A, Farokhzadian J, Forouzi MA, Shahraki SK. The effect of positive thinking training on anxiety and happiness among older adults: a quasi-experimental study. J Educ Health Promotion. 2024;13(1):65. 10.4103/jehp.jehp_1799_22. 10.4103/jehp.jehp_1799_22 PMC10979770 38559474

[19] Hadian M, Fereidooni-Moghadam M, Maghsoodi J. The effect of teaching positivity skills on life satisfaction of families with patients with mental disorders. Educ Ethics Nurs. 2023;12(3–4):31–9. 10.22034/ETHIC.2023.712452.

[20] Green J, Thorogood N. (2018). Qualitative methods for health research (4th ed.) 56–67.

[21] Reeves B, Gaus W. Guidelines for reporting non-randomized studies. Complement Med Res. 2004;11(1):46–52. 10.1159/000080576. 10.1159/000080576 15353903

[22] Hoffmann T, Glasziou P, Boutron I, Milne R, Perera R, Moher D, Michie S. Better reporting of interventions: template for intervention description and replication (TIDieR) checklist and guide. Gesundheitswesen (Bundesverband Der Arzte Des Offentlichen Gesundheitsdienstes (Germany)). 2016;78(3):175–88. 10.1055/s-0041-111066. 26824401 10.1055/s-0041-111066

[23] Daniel W, Cross C. Biostatistics: a foundation for analysis in the health sciences. 11th ed. Hoboken: Wiley; 2018.

[24] Ayoub S. (2019). Positive thinking and its relationship to the level of happiness among university students: an applied study on Al-Furqan Islamic University and Islamic Africa (Doctoral dissertation, International University of Africa).

[25] Taherkhani Z, Kaveh M, Mani A, Ghahremani L, Khademi K. The effect of positive thinking on resilience and life satisfaction of older adults: a randomized controlled trial. Sci Rep. 2023;13(1):3478. 10.1038/s41598-023-30684-y. 36859479 10.1038/s41598-023-30684-y PMC9977771

[26] Sari E. Educational studies. World Books Cairo. 1986;2(5):162–3.

[27] Fateel M. The impact of psychological adjustment on private university students’ academic achievement: case study. Int J High Educ. 2019;8(6):184–91. 10.5430/ijhe.v8n6p184.

[28] Tseng CH, Sim D. Sample size planning for pilot studies. 2021. 10.48550/arXiv.2105.05483. .34373840

[29] Stanley N, Chinedu N, Ada O, Aguiyi C. Psychological adjustment among family caregivers of patients with mental disorders in federal neuropsychiatric hospitals in Nigeria. Clin Res. 2022;3(1):1–12. 10.35702/clinres.10003.

[30] Abedi Z, Alavi M, Ghazavi Z, Visentin D, Cleary M. Improving coping styles in family caregivers of psychiatric inpatients using planned behavior problem-solving training. J Nurs Res. 2020;28(1):70–82. 10.1097/jnr.0000000000000320. 10.1097/jnr.0000000000000320 30907769

[31] Barjoee L, Amini N, Keykhosrovani M, Shafiabadi A. Effectiveness of positive thinking training on perceived stress, metacognitive beliefs, and death anxiety in women with breast cancer: perceived stress in women with breast cancer. Arch Breast Cancer. 2022;9(9):195–203. 10.32768/abc.202292195-203.

[32] Khurana R, Kumar N. A positive cognitive behavior therapy-based intervention with a caregiver of a patient with mental illness amidst the Covid-19 pandemic: a case study. Int J Cur Res Rev. 2021;13(12):239. 10.31782/IJCRR.2021.131242.

[33] Esmaeili A, Saeidi A, Mohammadi Y, Raeisoon M. The effect of group psychological training on the attitude of family caregivers of patients with schizophrenia. Heliyon. 2022;8(7). 10.1016/j.heliyon.2022.e09817. 10.1016/j.heliyon.2022.e09817 PMC9304719 35874056

[34] Haroon H, Bahiraei M. The effectiveness of positivism training on the life satisfaction and character strengths in the elderly men. Aging Psychol. 2020;6(2):179–89. 10.22126/JAP.2020.5647.1460.

[35] Bilicha P, Nashori F, Sulistyarini I. Positive thinking training for improving self-acceptance of children in a correctional facility. Jurnal Ilmiah Psikologi Terapan. 2022;10(2):89–93. 10.22219/jipt.v10i2.16557.

[36] Zoromba M, El-Gazar H, Salah A, El-Boraie H, El-Gilany A, El-Monshed A. Effects of emotional intelligence training on symptom severity in patients with depressive disorders. Clin Nurs Res. 2023;32(2):393–405. 10.1177/10547738221074065. 35114809 10.1177/10547738221074065

[37] El-Azzab S, Ali M, Ahmed A. Efficiency of nursing intervention on psychological adjustment, perfectionism and symptoms among patients with obsessive-compulsive disorder. Int Egypt J Nurs Sci Res. 2023;3(2):796–815. 10.21608/ejnsr.2023.283290.

[38] Jiang Y, Yin F, Lv Z, Hou H, Yang B, Liu Q, Wang X. Effect of the caregivers-to-caregivers training program on informed caregivers of persons with mental disorders: a pilot study. Int J Soc Psychiatry. 2024;70(2):289–97. 10.1177/00207640231207572. 37947259 10.1177/00207640231207572

[39] Sebaie S, Aziz M, Atia S. Positive thinking, resourcefulness skills, and future anxiety among the caregivers of children with intellectual disability: an intervention study. Middle East Curr Psychiatry. 2024;31(1):24. 10.1186/s43045-024-00412-x.

[40] Sari A, Duman Z. Effects of the family support and psycho-education program based on the Calgary family intervention model on the coping, psychological distress, and psychological resilience levels of the family caregivers of chronic psychiatric patients. Arch Psychiatr Nurs. 2022;41(1):1–10. 10.1016/j.apnu.2022.07.014. 36428035 10.1016/j.apnu.2022.07.014

[41] Almeida TC, Ifrim IC. Psychometric properties of the positive thinking skills scale (PTSS) among Portuguese adults. Behav Sci. 2023;13(5):357–68. 10.3390/bs13050357. 37232594 10.3390/bs13050357 PMC10215308

[42] Abdel-Aziz R, Abdel-Aziz H, Kotb F, Zaki S. Effect of Psycho-educational program on social functioning among schizophrenic patients. Minia Sci Nurs J. 2023;13(1):126–34. 10.21608/MSNJ.2023.217139.1069.

[43] Clari R, Headley J, Egger J, Swai P, Lawala P, Minja A, Baumgartner JN. Perceived burden and family functioning among informal caregivers of individuals living with schizophrenia in tanzania: a cross-sectional study. BMC Psychiatry. 2022;22(1):1–12. 10.1186/s12888-021-03560-0. 34983438 10.1186/s12888-021-03560-0 PMC8728903

[44] Zhou Z, Wang Y, Feng P, Li T, Tebes JK, Luan R, Yu Y. Associations of caregiving knowledge and skills with caregiver burden, psychological well-being, and coping styles among primary family caregivers of people living with schizophrenia in China. Front Psychiatry. 2021;12:631420. 10.3389/fpsyt.2021.631420. 34122169 10.3389/fpsyt.2021.631420 PMC8187614

[45] Fannick E. Role of resilience, quality of life, and social problem-solving in caregivers of patients living with dementia. *PCOM Psychol Dissertations*. 2024; 656. https://digitalcommons.pcom.edu/psychology_dissertations/656

[46] Liu C, Marino VR, Howard VJ, Haley WE, Roth DL. Positive aspects of caregiving in incident and long-term caregivers: role of social engagement and distress. Aging Ment Health. 2023;27(1):87–93. 10.1080/13607863.2021.2000935. 34749554 10.1080/13607863.2021.2000935 PMC9126189

