# Supplementary material for: Effect of a positive thinking skills training program on psychological adjustment among psychiatric patients’ caregivers
Source: BMC Psychiatry. 2025 Oct 23;25:1018. doi: 10.1186/s12888-025-07276-3 (PMC12548114; doi:10.1186/s12888-025-07276-3)
Supplement: Supplementary file 1 — Supplementary Material 1 [file 12888_2025_7276_MOESM1_ESM.docx]

**The program sessions were applied as follows:**

- **Session 1:** titled **"**Introduction and direction of the program". It involved welcome, establish interaction relationship between the researcher and the caregivers of psychiatric patients through utilize ice breaking as a communication technique. Help the caregivers to identify the nature the program including number of sessions, dates, the duration of each session. Emphasize the importance of attending the sessions and active participation.
- **Session 2:** "The meaning of psychological adjustment". It included the definition of psychological adjustment, its dimensions, importance, key factors affecting psychological adjustment and clarify its resulting effects on mental health.
- **Session 3:** " The concept of positivity" . It was provided a details information about concept of positive thinking, importance of positive thinking. Explain the differences between a negative and a positive thinker person.
- **Session 4:** "Being optimistic and living positively". The researcher teach the psychiatric patients’ caregivers how to practice problem solving strategies as a way to deal with daily stressors. Teach the caregivers to respect the positive aspects of their life and practice gratitude through written down good events of the day, positive situations, success, happiness and create of appreciation for the good memories in their lives (gratitude) and record their positive feeling.
- **Session 5:** " Challenging negative thoughts and practicing positive self-talk". The researcher train the caregiver to overcome (challenging) negative thoughts through revision of negative beliefs and using constructive language by noticing their thoughts and making a conscious effort to re-shape and replace these negative thoughts with more positive ones. As well as, teach the caregiver how to practice positive self-talk.
- **Session 6:** "Create a positive social relationship with other". The researcher motivate the psychiatric patients’ caregivers to surround themselves with positive people and loving them. Explain the role of positive relationships in life.
- **Session 7:** " Relaxation technique". The researcher train the psychiatric patients’ caregivers a four relaxation techniques for stress management include; deep breathing, imagination, deep muscle relaxation and mindfulness meditation through using video and role playing. Additionally, applying thought stopping method to enhance the positive thinking.
- **Session 8:** " Enjoyable and a fruitful life". Train the studied caregivers the ways to enter laughter into their life, how to build self-confidence and self- esteem. Establish healthy life style as good habits of practicing the exercise.
